# Risk Scores Based on Six Survival-Related RNAs in a Competing Endogenous Network Composed of Differentially Expressed RNAs Between Clear Cell Renal Cell Carcinoma Patients Carrying Wild-Type or Mutant Von Hippel–Lindau Serve Well to Predict Malignancy and Prognosis

**DOI:** 10.3389/fonc.2021.726671

**Published:** 2021-10-25

**Authors:** Rui Zhu, Xiezhao Li, Zhiduan Cai, Siyang Liang, Yaoji Yuan, Yuyu Xu, Dehui Lai, Haibo Zhao, Weiqing Yang, Jun Bian, Leyuan Liu, Guibin Xu

**Affiliations:** Department of Urology, The Fifth Affiliated Hospital of Guangzhou Medical University, Guangzhou, China

**Keywords:** ccRCC, ceRNA network, prognosis, risk score, VHL

## Abstract

Clear cell renal cell carcinoma (ccRCC) carrying wild-type Von Hippel–Lindau (VHL) tumor suppressor are more invasive and of high morbidity. Concurrently, competing endogenous RNA (ceRNA) network has been suggested to play an important role in ccRCC malignancy. In order to understand why the patients carrying wild-type VHL gene have high degrees of invasion and morbidity, we applied bioinformatics approaches to identify 861 differentially expressed RNAs (DE-RNAs) between patients carrying wild-type and patients carrying mutant VHL from The Cancer Genome Atlas (TCGA) database, established a ceRNA network including 122 RNAs, and elected six survival-related DE-RNAs including Linc00942, Linc00858, RP13_392I16.1, hsa-miR-182-5p, hsa-miR-183-5p, and PAX3. Examining clinical samples from our hospital revealed that patients carrying wild-type VHL had significantly higher levels of all six RNAs than those carrying mutant VHL. Patients carrying wild-type VHL had significantly higher risk scores, which were calculated based on expression levels of all six RNAs, than those carrying mutant VHL. Patients with higher risk scores had significantly shorter survival times than those with lower risk scores. Therefore, the risk scores serve well to predict malignancy and prognosis.

## Introduction

Kidney cancer is one of the top 10 most common tumors, the second-ranked malignant tumor in urologic system, and contributes 2% to 3% of the human malignant tumors worldwide (1, 2). There are 400,000 new cases of kidney malignant tumors of which 175,000 lead to death per year (3). Among patients with renal cancer at early stage and even some of renal cancer patients who do not have any specific symptom regardless of the stage of the disease, only about 30% of patients diagnosed with renal cancer was found to be at initial stage of malignancy, but the other 70% were found to be already at late stage of malignancy (4, 5). About 90% of the cases with kidney cancers are those with renal cell carcinomas, of which 70% are with clear cell renal cell carcinomas (ccRCCs), which are either hereditary or sporadic (6). Among many genetic factors that were found to be related to ccRCC, the inactivation of Von Hippel–Lindau (VHL) tumor suppressor gene occurs in 80% of patients and is the most common event (7–9), leading to the interruption of both vascular endothelial growth factor (VEGF) and mammalian target of rapamycin (mTOR) signaling pathways, which serve as the targets of most of the drugs currently used in clinical practice (10, 11). Although the inactivation of VHL gene alone is not sufficient to cause tumors (12, 13), a study of ccRCC patients from Germany suggested that low expression of VHL was identified as a risk factor for worse overall survival (OS) (14). However, it was reported that there is no difference in survival between patients carrying wild-type or mutant VHL or between patients with different levels of either VHL protein or messenger RNA (mRNA), and patients with nonsense mutations in exon 1 have a worse prognosis based on a study of ccRCC patients from Brazil (15), suggesting that loss of VHL function is directly related to the prognosis of ccRCC patients. A recent study indicated that about 40%–60% of patients with sporadic ccRCC carry wild-type VHL, and such tumors are more invasive and lead to a dramatic reduction of survival rates as compared with those carrying mutant VHL (16, 17). Therefore, it is more urgent to identify other factors that are closely associated with patients’ survival in addition to VHL mutation.

Long non-coding RNA (lncRNA) is a type of transcript with more than 200 nucleotides that does not encode a protein (18). It was initially considered to be “noise” generated by genome transcription without biological functions. More and more evidences show that lncRNA is involved not only in various physiological processes, such as cell proliferation, differentiation, and apoptosis, but also in pathological processes of various diseases (18). The long intergenic non-coding RNA (lincRNA) is the most common form of lncRNA. LncRNA is reported to be related to a variety of tumor diseases such as liver cancer (19), gastric cancer (20), oral squamous cell carcinoma (21), bladder cancer (22), prostate cancer (23), and kidney cancer (24). MicroRNAs (miRNAs) are small single-stranded non-coding molecules of about 22 nucleotides long and target the 3′UTR or CDS of mRNAs to form RNA-Induced Silencing Complexes (RISCs) to induce the degradation of the correspondent mRNA (25). Circular RNA (circRNA) is homologous to its target gene sequence and may act as a molecular sponge. Since lncRNA and mRNA share sequence homology, it is also possible to combine miRNA with lncRNA or circRNA to change expression levels of their downstream target genes. Therefore, miRNAs, lncRNAs, mRNAs, and other non-coding RNAs form a large-scale network of competing endogenous RNAs (ceRNAs), which are currently considered to be involved in the regulation of a variety of tumor diseases and play an important role in different stages of tumorigenesis (26).

Here, we constructed a ceRNA network after the sets of differentially expressed lncRNAs (DE-lncRNAs), miRNAs (DE-miRNAs), and mRNAs (DE-mRNAs) between patients carrying wild-type and those carrying mutant VHL, which were common in multiple database, were identified. Further identification of six survival-related RNAs including Linc00942, Linc00858, RP13_392I16.1, hsa-miR-182-5p, hsa-miR-183-5p, and PAX3, in the ceRNA network led to the building of a model in which risk scores were successfully applied to predict malignancy and prognosis of ccRCC.

## Materials and Methods

### Selection of Patient Data in The Cancer Genome Atlas Database

**Figure 1:**
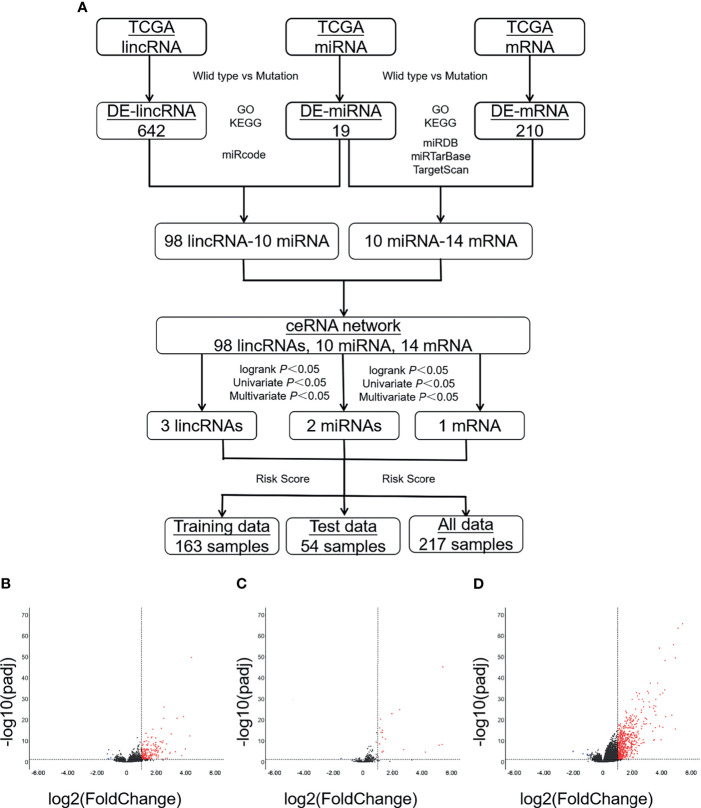
A large number of DE-RNAs are identified between patients carrying either wild-type or mutant VHL. **(A)** A diagram showing the workflow of the study. **(B–D)** Volcanic maps showing the distribution of DE-lncRNAs **(B)**, DE-miRNAs **(C)**, or DE-mRNAs **(D)**. Red, blue, and black dots represent those RNAs that are significantly upregulated, significantly downregulated, and not significantly changed, respectively. VHL, Von Hippel–Lindau; DE-RNAs, differentially expressed RNAs; DE-lncRNAs, differentially expressed long non-coding RNAs; DE-miRNAs, differentially expressed microRNAs; DE-mRNAs, differentially expressed messenger RNAs.

The selection of patient data in The Cancer Genome Atlas (TCGA) database was performed following the workflow as shown (**Figure 1A**). The expression data (read count) of ccRCC patients in TCGA were downloaded and integrated using R’s GDCRNATools package. The sample size for mRNA and lncRNA was 530, and miRNA was 516. The information about mutation of those samples was from cBioPortal (http://www.cbioportal.org/). The information about OS of those samples was from xenabrowser (https://xenabrowser.net/). Since RNA sequencing data were obtained directly from TCGA, approval by any ethics committee was not required.

### Identification of Differentially Expressed Long Non-Coding RNAs, Differentially Expressed MicroRNAs, and Differentially Expressed Messenger RNAs Between Clear Cell Renal Cell Carcinoma Patients Carrying Wild-Type or Mutant Von Hippel–Lindau

Among the 530 ccRCC samples with information of mRNA and lncRNA expression, samples of 446 patients were genotyped as either wild type (223 samples) or mutant (223 samples). Among the 516 samples with information of miRNA, samples of 220 patients were genotyped as wild-type, and 221 patients were genotyped as mutant VHL. The RNA read count of samples carrying wild-type and mutant VHL was standardized and analyzed with the DESeq2 package. DE-lncRNAs, DE-miRNAs, and DE-mRNAs were elected with |log2foldchange| > 1 and Adj.*p.*val < 0.05 as a threshold according to the method of Benjamini–Hochberg.

### Construction of Competing Endogenous RNA Regulation Network

The interaction between lncRNA and miRNA came from miRcode database (http://www.mircode.org/). The interaction between miRNA and mRNA came from miRDB (http://mirdb.org/), miRTarBase (http://mirtarbase.mbc.nctu.edu.tw/php/index.php), and TargetScan (http://www.targetscan.org/vert_72/) databases. In order to improve the reliability of the results, we selected the intersection of DE-miRNA–DE-mRNA interaction pairs found in the three databases as candidate genes to construct the ceRNA network. Finally, we used Cytoscape software to visualize the ceRNA network.

### Enrichment Analyses of the Competing Endogenous RNAs

The Gene Ontology (GO) molecular function enrichment and Kyoto Encyclopedia of Genes and Genomes (KEGG) pathway enrichment of DE-mRNA in the ceRNA network were analyzed with R’s ClusterProfiler package. *p* < 0.05 was considered statistically significant.

### Survival Analysis of Differential RNAs in the Competing Endogenous RNA Regulation Network

The expression level of each DE-lncRNA, DE-miRNA, and DE-mRNA in the ceRNA network in each patient was classified into either high- or low-expression group with their median as a threshold. The survival curve for each group was plotted through the Kaplan–Meier (K–M) survival analysis. *p* < 0.05 was considered statistically significant.

### Univariate and Multivariate Cox Regression Analyses

Univariate and multivariate Cox regression analyses were used to screen the survival-related differentially expressed RNAs (DE-RNAs) between patients carrying wild-type and those carrying mutant VHL to eliminate confounding factors, reduce the number of DE-RNAs, and calculate a hazard ratio (HR) and a 95% confidence interval (95% CI) for each variable. Genotypes with an HR greater than 1 or less than 1 and a Wald test *p*-value less than 0.05 were considered to be the ones that significantly affected patient survival.

### External Verification of Survival-Related RNAs With Clinical Tissue Samples

Tissue samples were collected from 21 postoperative ccRCC patients who underwent surgery but did not receive any anti-tumor radiotherapy or chemotherapy before surgery at The Fifth Affiliated Hospital of Guangzhou Medical University from January 2018 to May 2020. All samples were frozen and stored in liquid nitrogen immediately after sampling and subjected to DNA sequencing to determine if their VHL gene was wild type or mutant. The expression levels of VHL protein in the samples were determined by immunohistochemistry analyses with a polyclonal antibody against VHL protein (Cat# 16538-1-AP, from ProteinTech^®^). The expression levels of target RNAs in the samples were verified by qRT-RCR. Tumor samples were staged according to the 2010 American Joint Committee on Cancer (AJCC) TNM stage and clinical stage system, and their histopathological grades were determined with the Furman grade method. The study was approved by the Ethics Committee of The Fifth Affiliated Hospital of Guangzhou Medical University. Patients and their family members had been fully informed, and signed consent forms indicated that their samples would be used for scientific research.

### Establishment of a Prognostic Risk Score System and Clinical Correlation Analysis for Differentially Expressed RNAs in the Competing Endogenous RNA Regulation Network

A risk score system was established using the DE-RNAs selected by multivariate Cox regression analysis. To predict the patient’s prognosis, risk scores were calculated with the formula “Risk score = ∑(βi * Xi)” in which “i” was the number of characteristic genes, “β” the correlation coefficient of mRNA in the multivariate Cox regression analysis, and X the levels of gene expression after log2 conversion. Pearson’s chi-squared test and Fisher’s exact test were applied to analyze the correlation between patient’s risk score and clinicopathological characteristics in TCGA data set. *p* < 0.05 was considered to be statistically significant. The t-test was used to compare the difference of risk scores between clinical features that were significantly related to OS. *p* < 0.05 was considered to be statistically significant.

*Gene Set Enrichment Analysis* (GSEA) was applied to analyze the signaling pathways associated with patients carrying wild-type VHL and high risk score. The gene expression data from patients carrying high risk score and low risk score in the c2.cp.kegg.v7.0.symbols.gmt database were subjected to estimate the normalized enrichment scores (NESs), *p*-values, and *q*-values after false discovery rates (FDRs) were adjusted for each signal pathway with the GSEA software.

## Results

### Multiple Differentially Expressed Long Non-Coding RNAs, Differentially Expressed MicroRNAs, and Differentially Expressed Messenger RNAs Are Identified Between Patients Carrying Either Wild-Type or Mutant Von Hippel–Lindau

We analyzed the levels of lncRNAs, miRNAs, and mRNAs between ccRCC patients carrying wild-type and those carrying mutant VHL in TCGA database; displayed the distribution of those DE-RNAs in form of volcano map; and defined those RNAs with |log2foldchange| > 1 and Adj.*p.*val < 0.05 as DE-RNAs. A total of 642 DE-mRNAs (635 upregulated and seven downregulated), 210 DE-lncRNAs (205 upregulated and five downregulated), and 19 DE-miRNAs (18 upregulated and one downregulated) were identified (**Figures 1B–D**).

### A Competing Endogenous RNA Network Is Established

**Figure 2:**
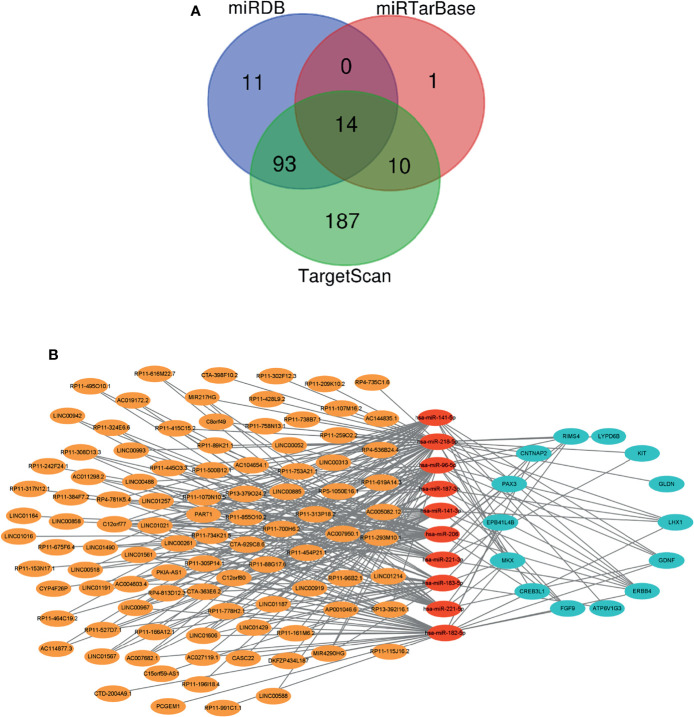
A ceRNA network is created. **(A)** A Venn diagram showing the miRNA–mRNA interaction as predicted based on three databases. **(B)** A diagram showing the established ceRNA network. Orange, red, and blue circles represent DE-lncRNAs, DE-miRNAs, and DE-mRNAs, respectively. Gray lines represent interactions among lncRNAs, miRNAs, and mRNAs. ceRNA, competing endogenous RNA; DE-lncRNAs, differentially expressed long non-coding RNAs; DE-miRNAs, differentially expressed microRNAs; DE-mRNAs, differentially expressed messenger RNAs.

We searched the miRcode database, identified 210 miRNAs that were targeted by DE-lncRNAs, and found interactions between 10 DE-miRNAs and 98 DE-lncRNAs. We searched the miRDB, miRTarBase, and TargetScan databases to identify interactions between miRNA–mRNA interaction and found 10 DE-miRNA and 14 DE-mRNA interactions (**Figure 2A**). Eventually, a ceRNA regulation network including 290 edges and 122 nodes (98 lncRNAs, 10 miRNAs, and 14 mRNAs) was established between patients carrying wild-type or mutant VHL as shown in **Figure 2B**.

### The Signaling Pathways Affected by Genes in Competing Endogenous RNA Network Are Predicted

**Figure 3:**
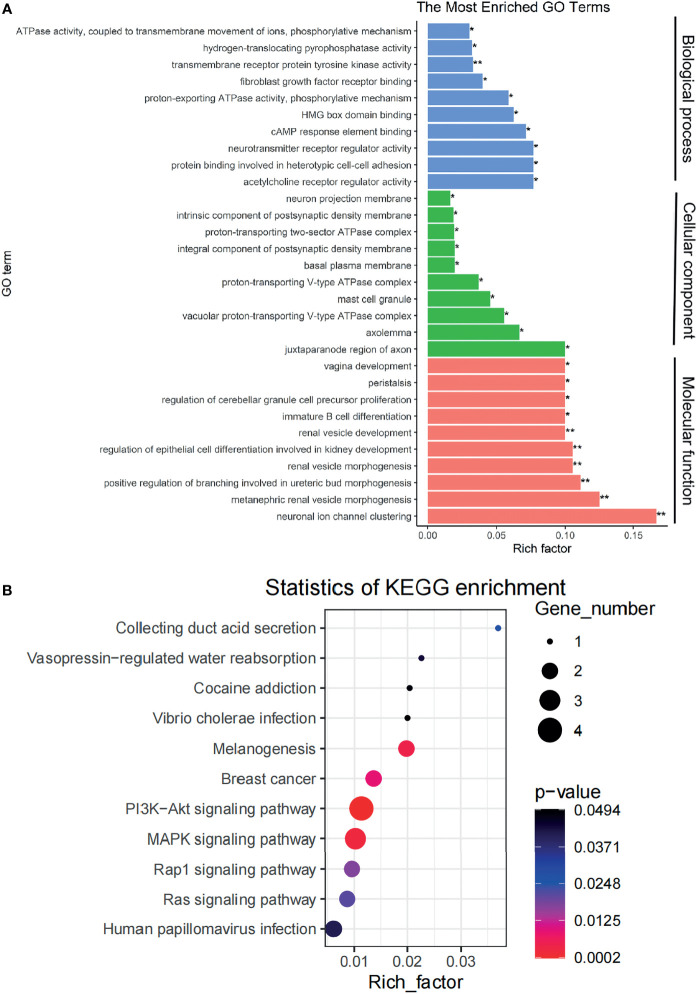
The signaling pathways affected by genes in the ceRNA network are identified. **(A)** A plot showing the signaling pathways affected by genes in the ceRNA network as detected *via* GO enrichment analyses. **(B)** A bubble chart showing the signaling pathways affected by genes in the ceRNA network as detected *via* KEGG pathways analysis. ceRNA, competing endogenous RNA; GO, Gene Ontology; KEGG, Kyoto Encyclopedia of Genes and Genomes. *p < 0.05, **p < 0.01.

We performed GO enrichment and pathway enrichment analysis on 14 differential genes in the ceRNA network and displayed the top 10 candidate signaling pathways based on the rich factors (the number of differential genes in the GO term divided by the total number of genes in the GO term). The biological processes were mainly related to some kinase activity, growth factor receptor binding, receptor regulatory activity, and so on; cell components were mainly involved in the formation of cell adhesion, synapses and axon membrane structures, and mast cell granules; and molecular functions were mostly related to the kidney functions such as renal vesicle development, epithelial cell differentiation during kidney development, kidney capsule morphogenesis, and renal vesicle morphogenesis (**Figure 3A**). KEGG enrichment search identified not only some kidney-related pathways such as collecting duct acid secretion and vasopressin-regulated water absorption but also some cancer-related pathways such as PI3K-AKT signaling pathway and MAPK signal pathway as shown in the bubble chart (**Figure 3B**).

### Survival-Related RNAs in Competing Endogenous RNA Network Are Identified Through Univariate and Multivariate Cox Regression Analyses

We estimated the significance of 98 lncRNAs, 10 miRNAs, and 14 mRNAs in the ceRNA network with OS of patients and identified 18 lncRNAs, four miRNAs, and three mRNAs, which significantly impacted OS either positively or negatively (**Table 1**). Univariate regression analysis found that these RNAs were significantly related to the aggressiveness of disease. Multivariate Cox regression analysis found that LINC00942, LINC00858, RP13_392I16.1, hsa-miR-183-5p, hsa-miR-182-5p, and PAX3 were considered as independent factors that impacted the prognosis of patients (**Table 1**). Therefore, these six RNAs were selected to build the risk score system.

**Table 1:** Univariate and multivariate cox regression analysis of survival-related lncRNAs, miRNAs, and mRNAs.

CeRNA	Impact	LogrankP	Univariate				Multivariate			
			Beta	HR	95% CI	*p* Value	Beta	HR	95% CI	*p* Value
**LncRNA**										
Linc00313	−	0.02	0.55	1.70	1.10–2.70	0.02	0.30	1.35	0.75–2.44	0.31
Linc00488	−	0.02	0.57	1.80	1.10–2.80	0.02	−0.01	0.99	0.57–1.73	0.97
CYP4F26P	−	0.04	0.48	1.60	1.00–2.60	0.04	−0.15	0.86	0.49–1.51	0.60
Linc00858	−	0.00	0.73	2.10	1.30–3.30	0.00	0.61	1.83	1.04–3.23	**0.04**
AC007682	−	0.01	0.65	1.90	1.20–3.00	0.01	−0.22	0.80	0.45–1.45	0.47
RP13-392I16.1	+	0.01	−0.64	0.52	0.32–0.86	0.01	−0.70	0.50	0.27–0.90	**0.02**
RP11-302F12.3	−	0.00	0.65	1.90	1.20–3.00	0.01	0.29	1.34	0.78–2.29	0.29
RP11-259O2.2	−	0.03	0.52	1.70	1.00–2.80	0.04	−0.10	0.90	0.48–1.69	0.75
Linc01016	−	0.00	0.70	2.00	1.20–3.30	0.00	0.34	1.41	0.77–2.58	0.27
Linc00942	−	0.00	0.96	2.60	1.60–4.30	0.00	0.74	2.09	1.09–4.01	**0.03**
CTD-2004A9.1	+	0.00	−0.71	0.49	0.31–0.79	0.00	0.13	1.14	0.60–2.17	0.69
RP11-96B2.1	−	0.00	0.68	2.00	1.20–3.10	0.00	0.06	1.06	0.58–1.94	0.85
RP11-619A14.3	−	0.03	0.52	1.70	1.10–2.70	0.03	−0.08	0.92	0.50–1.70	0.79
C8orf49	−	0.02	0.62	1.90	1.10–3.10	0.02	−0.01	0.99	0.55–1.77	0.96
AC144835	−	0.01	0.64	1.90	1.20–3.10	0.01	0.05	1.05	0.58–1.90	0.87
Linc00261	−	0.03	0.51	1.70	1.00–2.60	0.03	0.20	1.22	0.69–2.17	0.49
RP4-536B24.4	−	0.00	0.67	2.00	1.20–3.10	0.01	0.33	1.40	0.76–2.56	0.28
RP13-379O24.2	−	0.01	0.62	1.90	1.20–2.90	0.01	0.22	1.24	0.70–2.22	0.46
**miRNA**										
hsa-miR-182-5p	−	0.03	0.51	1.70	1.00–2.60	0.03	−1.31	0.27	0.12–0.63	**0.00**
hsa-miR-183-5p	−	0.00	1.10	2.90	1.70–4.80	0.00	1.52	4.58	1.97–10.62	**0.00**
hsa-miR-218-5p	−	0.03	0.52	1.70	1.10–2.70	0.03	0.41	1.51	0.79–2.89	0.21
hsa-miR-221-3p	−	0.00	1.00	2.80	1.70–4.50	0.00	0.65	1.91	0.99–3.69	0.05
**mRNA**										
RIMS4	−	0.01	1.90	1.90	1.20–3.10	0.01	0.11	1.12	0.63–1.98	0.70
PAX3	−	0.00	2.00	2.00	1.30–3.20	0.00	0.65	1.92	1.11–3.34	**0.02**
CREB3L1	−	0.02	1.70	1.70	1.10–2.70	0.02	−0.52	0.60	0.30–1.19	0.14

HR, hazard ratio; CI, confidence interval; lncRNAs, long non-coding RNAs; miRNAs, microRNAs; mRNAs, messenger RNAs; ceRNA, competing endogenous RNA. Values in bold are with p < 0.05.

### The Survival-Related RNAs Detected in Databases Are Verified With External Clinical Samples

**Figure 4:**
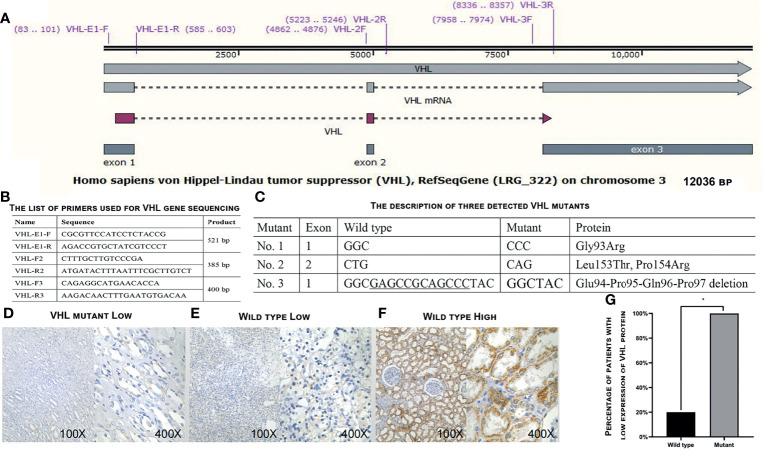
Determination of VHL gene expression in the 21 patients. **(A)** A diagram showing the structure of VHL gene and positions of primers used for gene sequencing. **(B)** The list of primers used to sequence VHL gene. **(C)** The description of three detected VHL mutants. **(D–F)** Representative images showing the low expression levels of VHL protein in patients carrying mutant VHL **(D)** or wild-type VHL **(E)** and high expression levels of VHL protein in patients carrying wild-type VHL as detected by immunohistochemical staining in ccRCC patients enrolled in our hospital. **(G)** A plot showing the percentage of patients with low expression levels of VHL protein in patients carrying wild-type or mutant VHL gene. VHL, Von Hippel–Lindau; ccRCC, clear cell renal cell carcinoma. *p < 0.05.

**Figure 5:**
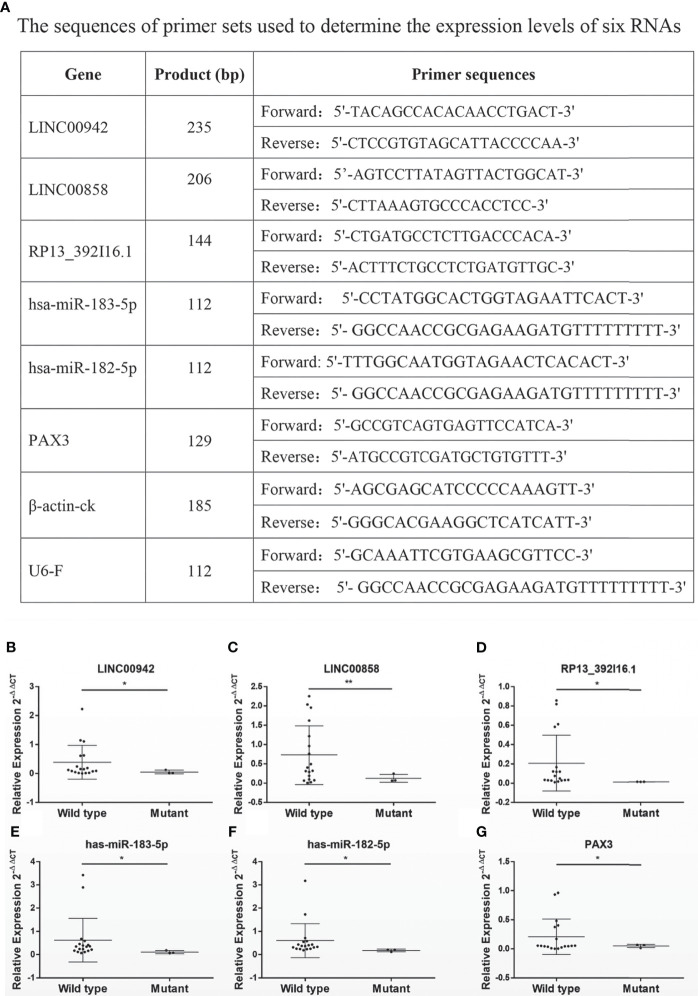
The survival-related RNAs detected in databases are verified with external clinical samples. **(A–G)** A list of primers used to determine **(A)** and scatter diagrams showing **(B–G)** the expression levels of survival-related RNAs LINC00942 **(B)**, LINC00858 **(C)**, RP13.392I16.1 **(D)**, hsa-miR-183-5p, hsa-miR-182-5p **(F)**, and PAX3 **(G)** as detected by qRT-PCR in 21 clinical patients (wild-type n = 18 and mutant n = 3). *p < 0.05, *p < 0.01.

A total of 21 ccRCC tissue samples were collected in our hospital and subjected to gene sequencing and immunohistochemistry analysis. Using three sets of primers covering the three exons of VHL gene (**Figures 4A, B**), we identified 18 samples carrying wild-type VHL and three samples carrying mutant VHL as shown in **Figure 4C**. The identification of high percentage of patients carrying wild-type VHL gene was a surprise to us and triggered us to investigate if the wild-type gene has been suppressed due to other epigenetic modifications. Patients carrying wild-type VHL gene expressed higher levels of VHL protein than those carrying mutant VHL (**Figures 4D–G**), suggesting that the expression of wild-type gene was not suppressed in general by other epigenetic events such as DNA methylation. The qRT-PCR analyses using primer sets listed (**Figure 5A**) demonstrated that levels of LINC00942, LINC00858, RP13_392I16.1, hsa-miR-183-5p, hsa-miR-182-5p, and PAX3 were significantly higher in patients carrying wild-type VHL than in patients carrying mutant VHL (**Figures 5B–G**). Therefore, the results obtained from databases were verified with external clinical samples.

### Risk Scores Are Significantly Different Between Patients with Different Clinical Characteristics

**Figure 6:**
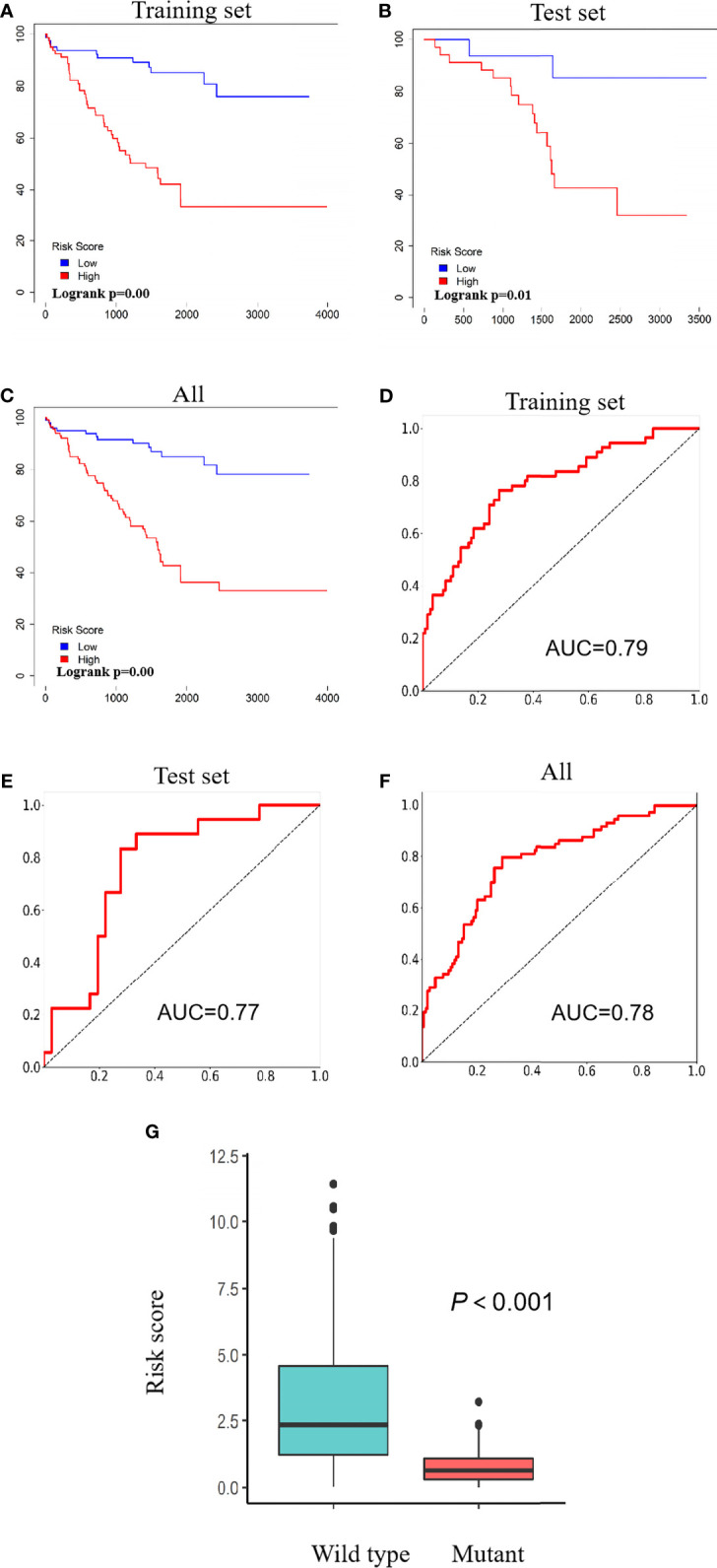
Risk scores predict patients’ survival. **(A–C)** Kaplan–Meier curves showing the relationship between risk scores and patients’ survival in the training set **(A)**, test set **(B)**, and all patients **(C)**. **(D–F)** ROC curves showing AUC values of the training set **(D)**, test set **(E)**, and all patients **(F)**. **(G)** A scatter diagram showing differences of risk scores between patients carrying wild type and those carrying mutant VHL. ROC, receiver operating characteristic; AUC, area under the curve; VHL, Von Hippel–Lindau.

Based on a ratio of 3 to 1, we divided 217 ccRCC patients carrying wild-type VHL into a training set (163 cases) and a test set (54 cases), attributed each patient in the training set with a risk score, and separated patients into a high-risk group (81 patients) and a low-risk group (82 patients) with the median of risk scores 1.77 as the threshold. Patients in the high-risk group exhibited significantly worse prognosis than those in the low-risk group in both training set and test set as well as in all patients (**Figure 6A–C**). Receiver operating characteristic (ROC) curve analysis detected an area under the curve (AUC) value of 0.79, 0.77, or 0.78 and further confirmed the results from survival analyses (**Figures 6D–F**). Patients carrying wild-type VHL had significantly higher risk scores than those carrying mutant VHL (**Figure 6G**). There was no significant difference in risk scores between different age groups, but patients at higher and advanced ccRCC classification stages exhibited significantly higher risk scores (**Table 2**).

**Table 2:** The analyses of correlation between risk scores and clinicopathological characteristics of patients in TCGA data set.

Clinical features	Case	Low risk	High risk	*p*	X ± S	*p*
Age						
<55	107	46	61		2.37 ± 2.91	
≥55	110	55	55	0.37	2.34 ± 3.02	0.94
Pathological T						
T1–T2	131	73	58		1.62 ± 2.61	
T2–T4	86	28	58	**0.00**	3.46 ± 3.13	**0.00**
Pathological M						
M0	179	91	88		2.03 ± 2.88	
M1	38	10	28	**0.01**	3.89 ± 2.88	**0.00**
Pathological N						
N0	105	56	49		1.98 ± 3.12	
N1	9	2	7	0.09	4.93 ± 3.94	**0.01**
Grade						
Grade 1–2	95	58	37		1.20 ± 2.33	
Grade 3–4	118	39	79	**0.00**	3.38 ± 3.07	**0.00**
Overall status						
Alive	144	87	57		1.35 ± 2.31	
Dead	73	14	59	**0.00**	4.33 ± 3.13	**0.00**
Neoplasm status						
Tumor free	128	75	53		1.49 ± 2.37	
With tumor	74	17	57	**0.00**	4.01 ± 3.08	**0.00**
Tumor stage						
I–II	120	70	50		1.43 ± 2.53	
III–IV	96	30	66	**0.00**	3.55 ± 3.04	**0.00**

TCGA, The Cancer Genome Atlas.

Values in bold are with p < 0.05.

### Analysis of Signaling Pathway in Clear Cell Renal Cell Carcinoma Patients Carrying Wild-Type Von Hippel–Lindau and High Risk Scores

We conducted GSEA and identified 24 signaling pathways such as the “OLFACTORY_TRANSDUCTION” and “INTESTINAL_IMMUNE_NETWORK_FOR_IGA_PRODUCTION,” which were enriched in the group of patients with high risk scores (**Table 3**).

**Table 3:** GSEA analysis of signaling pathways associated with patients with high risk scores.

Names of signaling pathways	NES	NOM *p*-Val	FDR *q*-Val
OLFACTORY_TRANSDUCTION	2.22	0.00	0.00
INTESTINAL_IMMUNE_NETWORK_FOR_IGA_PRODUCTION	2.12	0.00	0.00
P53_SIGNALING_PATHWAY	2.11	0.00	0.00
GLYCOSAMINOGLYCAN_BIOSYNTHESIS_CHONDROITIN_SULFATE	2.04	0.00	0.00
TASTE_TRANSDUCTION	2.00	0.00	0.00
CYTOKINE_CYTOKINE_RECEPTOR_INTERACTION	1.92	0.00	0.01
HOMOLOGOUS_RECOMBINATION	1.91	0.00	0.01
CELL_CYCLE	1.80	0.00	0.02
ECM_RECEPTOR_INTERACTION	1.76	0.00	0.02
DNA_REPLICATION	1.67	0.01	0.05
BASE_EXCISION_REPAIR	1.67	0.01	0.04
BASAL_CELL_CARCINOMA	1.62	0.01	0.06
SYSTEMIC_LUPUS_ERYTHEMATOSUS	1.61	0.01	0.06
HEMATOPOIETIC_CELL_LINEAGE	1.61	0.00	0.06
DILATED_CARDIOMYOPATHY	1.58	0.00	0.07
GLYCOSAMINOGLYCAN_DEGRADATION	1.56	0.03	0.08
HYPERTROPHIC_CARDIOMYOPATHY_HCM	1.54	0.01	0.09
HEDGEHOG_SIGNALING_PATHWAY	1.53	0.02	0.08
PATHOGENIC_ESCHERICHIA_COLI_INFECTION	1.52	0.02	0.09
NEUROACTIVE_LIGAND_RECEPTOR_INTERACTION	1.51	0.00	0.09
ARRHYTHMOGENIC_RIGHT_VENTRICULAR_CARDIOMYOPATHY_ARVC	1.48	0.02	0.10
PRIMARY_IMMUNODEFICIENCY	1.45	0.04	0.13
LEISHMANIA_INFECTION	1.45	0.03	0.12
JAK_STAT_SIGNALING_PATHWAY	1.34	0.03	0.23

GSEA takes NOM less than 0.05 and FDR q-val less than 0.25 to filter the results.

NES, normalized enrichment score; NOM p-val, credibility of the enrichment result; FDR q-val: p-value after multiple hypothesis correction; GSEA, Gene Set Enrichment Analysis; FDR, false discovery rate.

## Discussion

A ceRNA network has been proposed to play important roles in ccRCC tumorigenesis and aggressiveness. Previous studies have revealed that the LINC01094 enhances the expression of miR-184 and inhibits the expression of SLC2A3, which suppresses the development of ccRCC (27). LINC01426 interacts with insulin-like growth factor 2 mRNA binding protein 1 (IGF2BP1) to enhance expression of CTBP1 in cytoplasm and promote the binding of CTBP1 to the promoter of miR-423-5p in the nucleus to recruit HDAC2 to synergistically inhibit the expression levels of miR-423-5p, leading to an elevation of expression levels of FOXM1 and a promotion of proliferation and migration of ccRCC cells (28). The ceRNA network constructed in this study yielded 98 DE-lncRNAs, 10 DE-miRNAs, and 14 DE-mRNAs. Analyses of the 14 DE-mRNAs revealed their involvement in functions related to kidney. Further analysis of the relationship of DE-RNAs in the ceRNA network with patients’ survival rates led to the identification of LINC00942, LINC00858, RP13_392I16.1, hsa-miR-182-5p, hsa-miR-183-5p, and PAX3, which were used to calculate risk scores for each individual patient as independent factors to successfully predict the malignancy and prognosis of patients.

Three lncRNAs was identified to be survival-related ceRNAs. Currently, there is no report related to any role of RP13_392I16.1 yet, so this lncRNA needs to be further studied. LINC00942 was reported to promote METT14-mediated M6A methylation and regulate the expression and stability of its target gene CXCR4 and CYP1B1 during the initiation and progression of breast cancer (29). In another report, LINC00942 was identified as one of seven lncRNAs in an lncRNA signature related to immune cell infiltration and immune checkpoint blockade of immunotherapy-related molecules and may serve as a prognostic biomarker of hepatocellular carcinoma (30). Similarly, LINC00942 was identified as one of four immune-related lncRNAs to predict prognosis and immunotherapy efficiency in bladder cancer patients (31). It also reported that LINC00942 was identified as one of the two components in a ceRNA network associated with gene number copy variation, which can be used to predict tumor response to drug treatment in patients with lung adenocarcinomas (32). Initially, LINC00858 was identified as a ceRNA impacting miR-422a to control the expression of kallikrein-related peptidase 4, and its high expression was found to be closely correlated to tumor progression of non-small cell lung carcinomas (33). It was reported to be one of the four lncRNAs delivered from database search that were further validated with clinical samples (34) and able to promote colorectal cancer by sponging miR-4766-5p (35) or miR-22-3P (36). Literatures published recently revealed that LINC00858 was found to play key roles in multiple types of cancers including Wilms’ tumor (37), ovarian cancer (38), colon cancer (39), gastric cancer (40), hepatocellular carcinomas (41), and osteosarcomas (42).

Both hsa-miR-182-5p and hsa-miR-183-5p belong to the miR-183/96/182 family because of their sequence homology and function similarity (43). They were found to be among the 10 miRNA signatures that significantly distinguish cancer tissues from adjacent normal tissues from patients with ovarian cancer (44), the five miRNAs associated with tumorigenesis in lung adenocarcinomas (45), the top three miRNAs that target the largest number of genes in atypical endometrial hyperplasia (46), and the five miRNAs that show optimal diagnostic biomarkers for hepatocellular carcinomas (47). The levels of hsa-miR-182-5p are elevated in tumor tissues from patients with ovarian cancer and may predict poor prognosis (48). hsa-miR-182-5p was reported to be associated with resistance of breast cancer cell lines to anti-tumor drug veliparib (49). The significance of hsa-miR-183-5p with additional 10 genes in survival of ccRCC patients has been reported, and high expression of hsa-miR-183-5p predicts a reduced OS rate and poor prognosis (50). It was reported that hsa-miR-182-5p is reduced in renal cancer tissues and cell lines and regulates the expression of *DLL4* gene, which causes change of tumor microenvironment and tumor inhibition (51).

PAX3 gene (paired box gene 3) encodes a member of PAX family of transcription factors whose target genes impact proliferation, survival, differentiation, and motility (52). Rhabdomyosarcoma is one of the typical tumors in children and adolescence with a poor prognosis and an OS rate of only 20%–40% (53). PAX3-FOXO1 or PAX3-FKHR, a specific fusion gene that resulted from chromosomal translocation, is an important factor in the occurrence and development of rhabdomyosarcoma (54, 55). Such fusion gene was also reported to be associated with other types of cancers such as melanoma (56) and biphenotypic sinonasal sarcoma, a low-grade spindle cell sarcoma that affects middle-aged adults (57). PAX3 is highly expressed in prostate cancer tissues and cell lines and promotes the progression of prostate cancer by inhibiting the TGF-β/SMAD signal axis (58). Although there is no report about PAX3 in ccRCC, PAX3 gene is differentially hyper-methylated in chromophobe renal cell carcinomas compared with renal oncocytomas, a benign kidney neoplasm (59).

As discussed above, the relation between VHL mutation and patient survival is different in different reports. We identified LINC00942, LINC00858, RP13_392I16.1, hsa-miR-182-5p, hsa-miR-183-5p, and PAX3 as survival-related DE-RNAs between patients carrying wild-type or mutant VHL and were further used to calculate risk scores for each individual patient. The identified RNAs may not directly impact the VHL function. Because each of these DE-RNAs has already been shown to play significant roles in the tumorigenesis and aggressiveness of other types of cancers as discussed above, it is predicted that the risk scores may serve well as factors independent to VHL gene status to predict the malignancy and prognosis of ccRCC patients in the future.

## Data Availability Statement

The raw data supporting the conclusions of this article will be made available by the authors, without undue reservation.

## Ethics Statement

The studies involving human participants were reviewed and approved by The Ethics Committee of The Fifth Affiliated Hospital of Guangzhou Medical University. The patients/participants provided their written informed consent to participate in this study.

## Author Contributions

RZ, XL, ZC, LL, and GX have full access to all of the data in the study, take responsibility for the integrity of the data and the accuracy of the data analysis, and wrote the manuscript. SL, YY, YX, DL, HZ, WY, and JB contributed the reagents. All authors contributed to the article and approved the submitted version.

## Funding

This study is partially supported by the National Natural Science Foundation of China 81974392 and Natural Science Foundation of Guangdong Province 2018A030313087 to GX, the National Natural Science Foundation of China 82103359 to ZC, the Health Technology Project of Guangzhou City 20201A011106 and 20211A011103 to JB, and the National Natural Science Foundation of China 81772931 to LL.

## Conflict of Interest

The authors declare that the research was conducted in the absence of any commercial or financial relationships that could be construed as a potential conflict of interest.

## Publisher’s Note

All claims expressed in this article are solely those of the authors and do not necessarily represent those of their affiliated organizations, or those of the publisher, the editors and the reviewers. Any product that may be evaluated in this article, or claim that may be made by its manufacturer, is not guaranteed or endorsed by the publisher.
